# UniProt: a hub for protein information

**DOI:** 10.1093/nar/gku989

**Published:** 2014-10-27

**Authors:** The UniProt Consortium

**Affiliations:** 1 European Molecular Biology Laboratory, European Bioinformatics Institute (EMBL-EBI), Wellcome Trust Genome Campus, Hinxton, Cambridge, CB10 1SD, UK; 2 SIB Swiss Institute of Bioinformatics, Centre Medical Universitaire, 1 rue Michel Servet, 1211 Geneva 4, Switzerland; 3 Protein Information Resource, Georgetown University Medical Center, 3300 Whitehaven Street North West, Suite 1200, Washington, DC 20007, USA; 4 Protein Information Resource, University of Delaware, 15 Innovation Way, Suite 205, Newark, DE 19711, USA

## Abstract

UniProt is an important collection of protein sequences and their annotations, which has doubled in size to 80 million sequences during the past year. This growth in sequences has prompted an extension of UniProt accession number space from 6 to 10 characters. An increasing fraction of new sequences are identical to a sequence that already exists in the database with the majority of sequences coming from genome sequencing projects. We have created a new proteome identifier that uniquely identifies a particular assembly of a species and strain or subspecies to help users track the provenance of sequences. We present a new website that has been designed using a user-experience design process. We have introduced an annotation score for all entries in UniProt to represent the relative amount of knowledge known about each protein. These scores will be helpful in identifying which proteins are the best characterized and most informative for comparative analysis. All UniProt data is provided freely and is available on the web at http://www.uniprot.org/.

## INTRODUCTION

We are at a critical point in the development of protein sequence databases. Continuing advances in next generation sequencing mean that for every experimentally characterized protein, there are now many hundreds of proteins that will never be experimentally characterized in the laboratory. In addition, there are new data types being introduced by developing high-throughput technologies in proteomics and genomics. The combination of both provides new opportunities for the life sciences and the biomedical domain. Therefore, it is crucial to identify experimental characterizations of proteins in the literature and to capture and integrate this knowledge into a framework in combination with high-throughput data and automatic annotation approaches to allow it to be fully exploited. UniProt facilitates scientific discovery by organizing biological knowledge and enabling researchers to rapidly comprehend complex areas of biology.

In brief, UniProt is composed of several important component parts. The section of UniProt that contains manually curated and reviewed entries is known as UniProtKB/Swiss-Prot and currently contains about half a million sequences. This section grows as new proteins are experimentally characterized (1). All other sequences are collected in the unreviewed section of UniProt known as UniProtKB/TrEMBL. This portion of UniProt currently contains around 80 million sequences and is growing exponentially. Although entries in UniProtKB/TrEMBL are not manually curated they are supplemented by automatically generated annotation. UniProt also makes available three sets of sequences that have been made non-redundant at various levels of sequences identity: UniRef100, UniRef90 and UniRef50 (2). The UniParc database is a comprehensive set of all known sequences indexed by their unique sequence checksums and currently contains over 70 million sequences entries (3). The UniProt database has cross-references to over 150 databases and acts as a central hub to organize protein information. Its accession numbers are a primary mechanism for accurate and sustainable tagging of proteins in informatics applications.

In this manuscript we describe the latest progress on developing UniProt. There are numerous challenges facing UniProt's goal to organize and annotate the universe of protein sequences. In particular, the great growth of microbial strain sequences has motivated us to create a new proteome identifier, which is described in more detail below. A central activity of UniProt is to curate information about proteins from the primary literature. In this paper we look at the annotation of enzymes with a focus on orphan enzyme activities. The UniProt database is used by thousands of scientists around the world every day and its website has been visited by over 400 000 unique visitors in 2013. We describe a complete redevelopment of the website based on a user experience design process below.

## PROGRESS AND NEW DEVELOPMENTS

### Growth of proteomes and other sequence data

**Figure 1.**
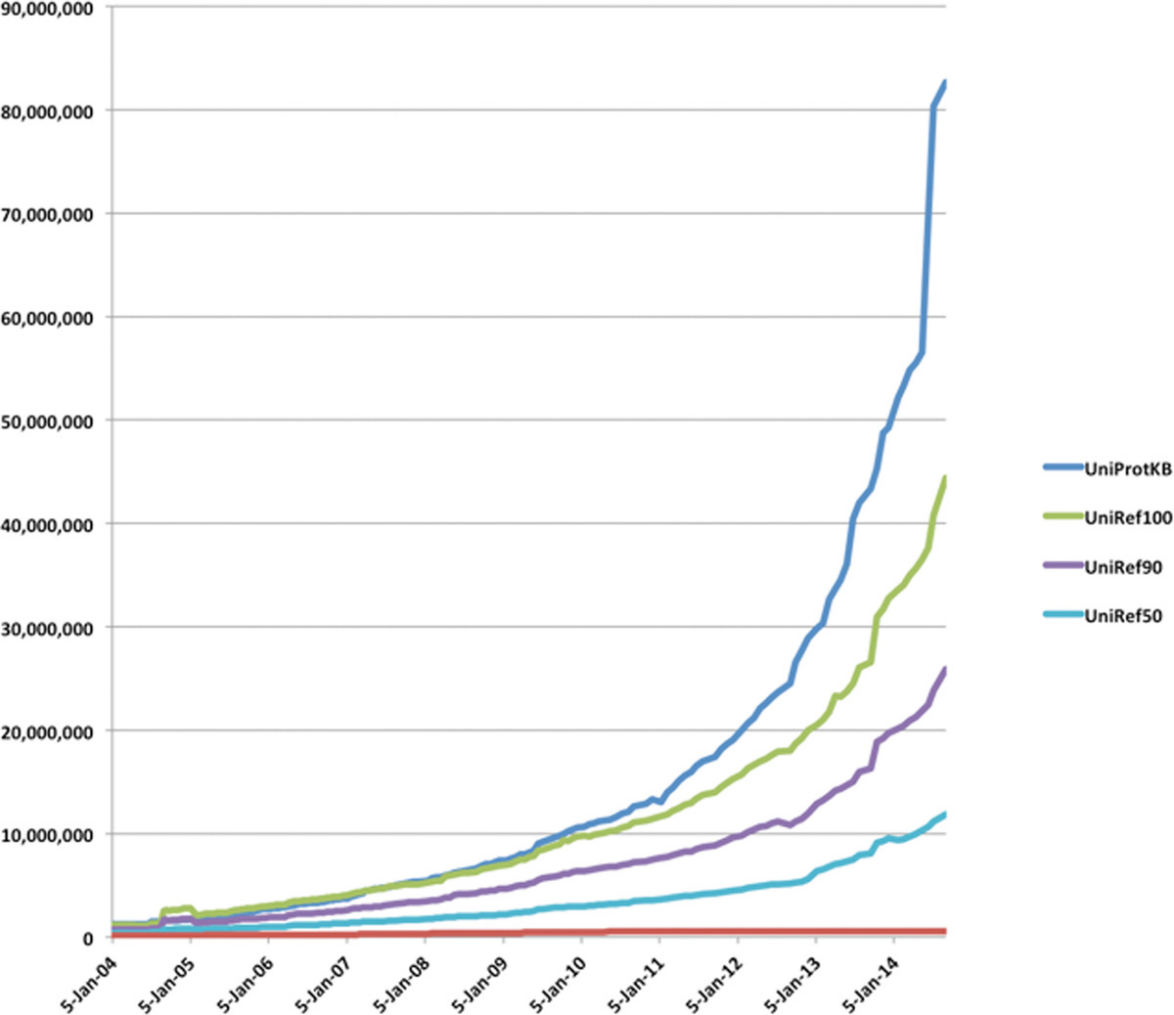
Growth of UniProt and UniRef databases.

**Figure 2.**
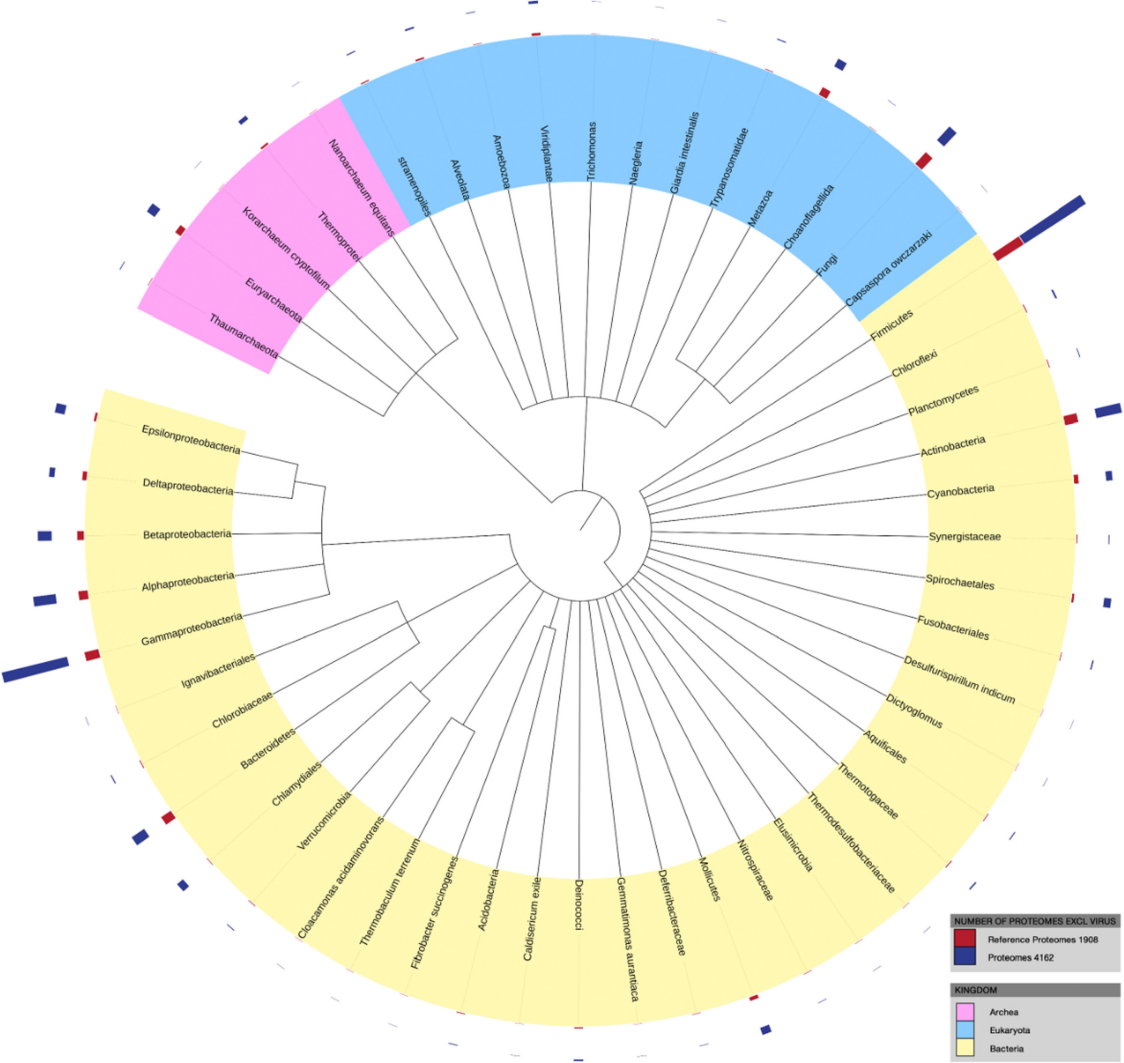
The distribution of proteomes and reference proteomes across the tree of life.

The number of protein sequences in UniProt continues to rise at an accelerated pace. As shown in Figure 1, during 12 releases from June 2013 to August 2014 the number of sequences in UniProt has doubled from 40.4 to 80.7 million records showing exponential growth. The UniRef databases, which cluster sequences at 100%, 90% and 50% identity illustrate the levels of sequence redundancy. From 2004 to 2014 the relative reduction in database size went from 5%/42%/70% to 54%/73%/88% for UniRef100, UniRef90 and UniRef50, respectively. Most of the growth in sequences is due to the increased submission of complete genomes to the nucleotide sequence databases (4). The majority of these genomes are derived from whole genome shotgun studies with bacterial genomes accounting for 80% of the data. In addition, there is an increase in submissions of multiple genomes for strains of the same organism or closely related species. While this wealth of protein information presents our users with new opportunities for proteome-wide analysis and interpretation, it also creates challenges in capturing, searching, preserving and presenting proteome data to the scientific community. To make room for new sequences we have increased our accession number format from 6 to 10 characters. Details of the new format are available at www.uniprot.org/help/accession_numbers. To help cope with these challenges in tracking and displaying proteomes we have reorganized our handling and display of proteomes and introduced a new proteome identifier that uniquely identifies the set of proteins corresponding to a single assembly of a completely sequenced genome. For users that prefer to use a single best-annotated proteome from a particular taxonomic group for their analysis, UniProt selects a proteome. UniProt reference proteomes are derived via consultation with the research community or computationally determined from proteome clusters (5) where the reference proteome is selected from the cluster by an algorithm that considers the best overall annotation score. Reference proteomes have been chosen to provide broad coverage of the tree of life and constitute a representative cross-section of the taxonomic diversity found within UniProt (Figure 2). There are currently 2290 reference proteomes selected. They are the focus of both manual and automatic annotation, aiming to provide the best annotated protein sets for the selected species. They include model organisms and other proteomes of interest to biomedical and biotechnological research. The protein sets for these species are generated in collaboration with the INSDC, Ensembl and RefSeq reference genomes. An example of a reference proteome can be found in the new proteome information page and proteome identifier http://www.uniprot.org/proteomes/UP000000803.

For users that prefer all versions and variants of a proteome, the non-reference proteomes will still be available in UniProt.

### Manual curation progress

Literature-based expert curation is a core UniProt activity. It provides high-quality annotation for experimentally characterized proteins across diverse protein families and taxonomic groups in the UniProtKB/Swiss-Prot section of UniProt. Although labour-intensive, the benefits of creating such a rich annotated data set are manifold, both for wet-lab researchers by providing an up-to-date knowledgebase containing experimental information, and computer scientists by providing high-quality training sets for development and enhancement of bioinformatics algorithms. Last but not least, it also serves as an essential source for the generation of automatic annotation for uncharacterized proteins, a key challenge in the era of next generation sequencing. The wealth of curation experience accumulated over the years within the consortium has created an expert team in this field. During 2013 we curated over 8400 papers and created over 3300 new UniProtKB/Swiss-Prot entries.

To provide the maximum amount of high-quality information to users, the choice of publications to curate is critical. We do not aim to curate all published papers but instead select a representative subset to provide a complete overview of available information according to well-established criteria using both literature surveillance and automatic systems (see (1) for a more detailed description). One important area of focus is the expert curation of enzymatic reactions. In light of the huge amounts of available sequence data, which offer a unique opportunity for new enzyme discovery, it is essential to provide a corpus of well-annotated characterized enzymes to facilitate the identification of new enzyme activities. By taking the example of expert curation of enzymes, we will detail how we prioritize proteins for curation, highlight annotation content and briefly describe some ongoing and future curation developments.

In recent years, specific emphasis has been put on the annotation of orphan enzymes, a group of enzymes that have been experimentally characterized but lack associated amino acid sequences (6,7). Identification of such enzymes can be difficult and we were helped by a recent publication reporting the identification of many orphan enzymes based on literature review and database searches (6). Relevant enzymes described in this publication have been prioritized for curation and this work is ongoing.

**Figure 3.**
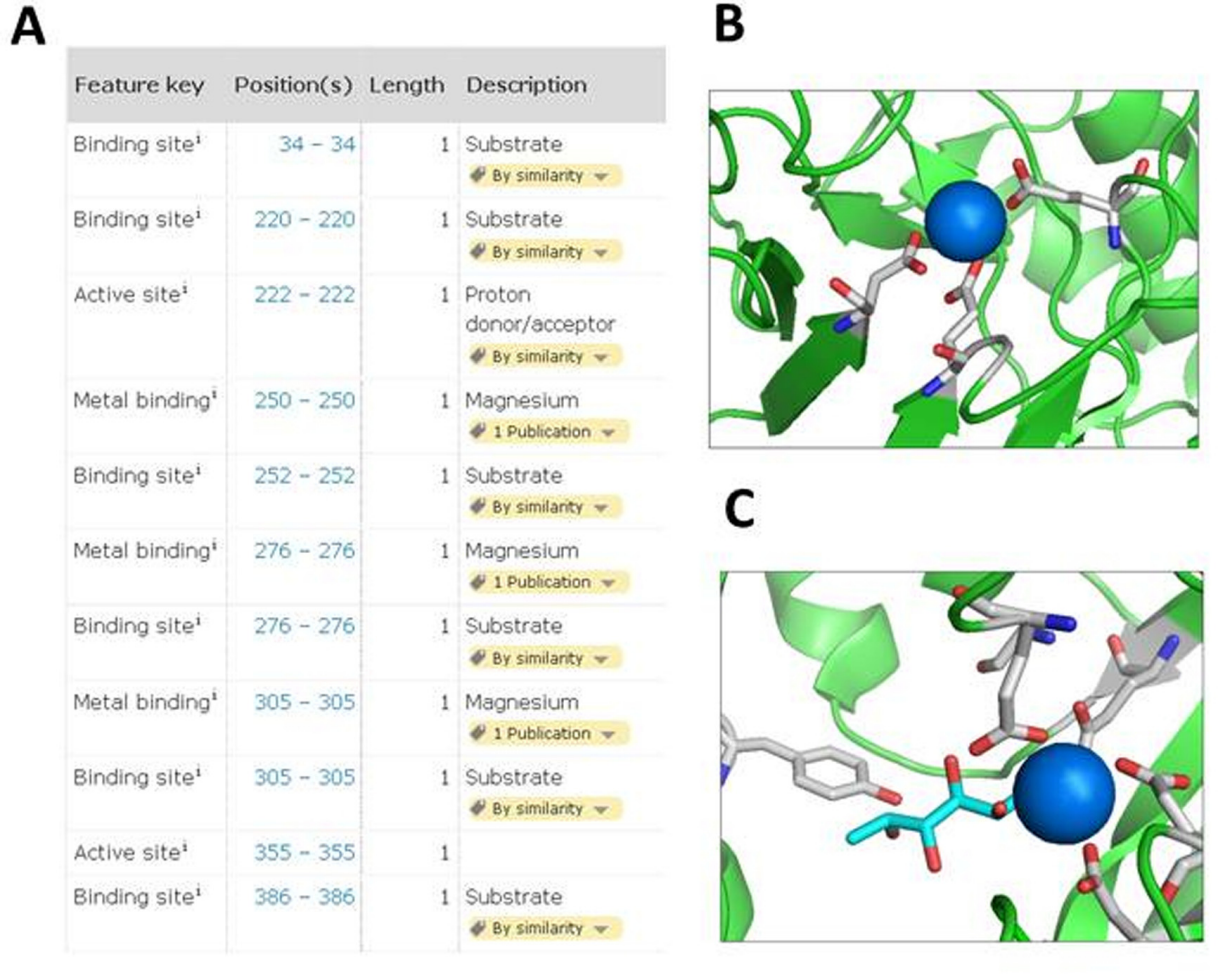
(A) Screenshot of the sequence annotation section of human ENOSF1 entry (UniProt Q7L5Y1, http://www.uniprot.org/uniprot/Q7L5Y1). (B) Image of magnesium-binding sites of human ENOSF1 X-ray structure in complex with magnesium (blue) using PyMol (PDB accession 4A35). (C) Image of active site of *X. campestris* fuconate dehydratase X-ray structure in complex with magnesium (blue) and substrate (green) using PyMol (PDB accession 2HXT).

High priority is also given to previously uncharacterized enzymes in reference proteomes. A good example is provided by the human mitochondrial enolase superfamily member 1 protein (UniProt Q7L5Y1). The ENOSF1 gene coding for this protein was initially thought to code for an antisense RNA to thymidylate synthase, able to down-regulate TYMS activity (8,9). Its function remained largely unknown for many years until recently, with its identification as an L-fuconate dehydratase (10). L-fuconate dehydratase is involved in catabolism of L-fucose, a sugar that is part of the carbohydrates that are attached to cellular glycoproteins, and catalyzes the dehydration of L-fuconate to 2-keto-3-deoxy-L-fuconate. Interestingly, this enzyme had not been identified in eukaryotes before and was previously characterized in *Xanthomonas campestris* only (UniProt Q8P3K2) (11). Relevant publications have been read in detail and fully curated and all information from the various papers has been compiled into a concise but comprehensive report that provides a complete overview of the information available about this protein including information related to function, catalytic activity, cofactor, subcellular location and biophysicochemical properties. We have also added a CAUTION comment to warn users that the originally proposed function of an antisense RNA to thymidylate synthase is unclear. Sequence annotations are of particular interest for enzymes, the conservation (or non-conservation) of sites important for catalysis or substrate binding being essential to predict the function of uncharacterized enzymes or to predict substrate specificity of homologous enzymes. A lot of sequence annotation information is derived from 3D structures, leveraging information from the Protein Data Bank (PDB) in combination with information extracted from the papers. In the case of ENOSF1 (UniProt Q7L5Y1), a high-resolution crystal structure in complex with magnesium cofactor is available, allowing the annotation of cofactor binding sites on the sequence (10). A crystal structure in complex with magnesium and substrate is available for *X. campestris* fuconate dehydratase (UniProt Q8P3K2), allowing the reliable propagation of the substrate binding sites to the human entry, thanks to the high sequence conservation of around 50% between the two enzymes (Figure 3).

The knowledge collected is represented using standardized vocabularies to facilitate subsequent retrieval whenever possible. A number of annotation fields related to enzymes are structured in this way. The catalytic activity annotation field follows the recommendations of the Nomenclature Committee of the International Union of Biochemistry and Molecular Biology (IUBMB) and we actively participate in the creation of new Enzyme Commission (EC) numbers by submitting new reactions to the IUBMB when required. In addition, UniProt is a major contributor to the Gene Ontology (GO) (12) and manual curation of GO terms based on experimental data from the literature is part of the UniProt curation process. We are expanding the use of controlled vocabularies in a number of annotation fields. For example, we recently changed the cofactor comment from free-text to a structured comment and introduced the controlled vocabulary of the Chemical Entities of Biological Interest (ChEBI) ontology (13), improving the representation of chemical identifiers and making access to this information easier for users.

In the future, we also plan to enhance the representation of the catalytic activity field for the construction of metabolic models as the current representation of catalytic reactions has limitations in providing a comprehensive and precise description of all possible chemical reaction variants in all organisms. We plan to address this by making use of resources, such as the manually curated Rhea database of chemical reactions (14) which includes enzyme-catalyzed reactions, transport reactions and spontaneously occurring reactions and which uses the ChEBI ontology to describe these reactions.

### Automatic annotation progress

**Figure 4.**
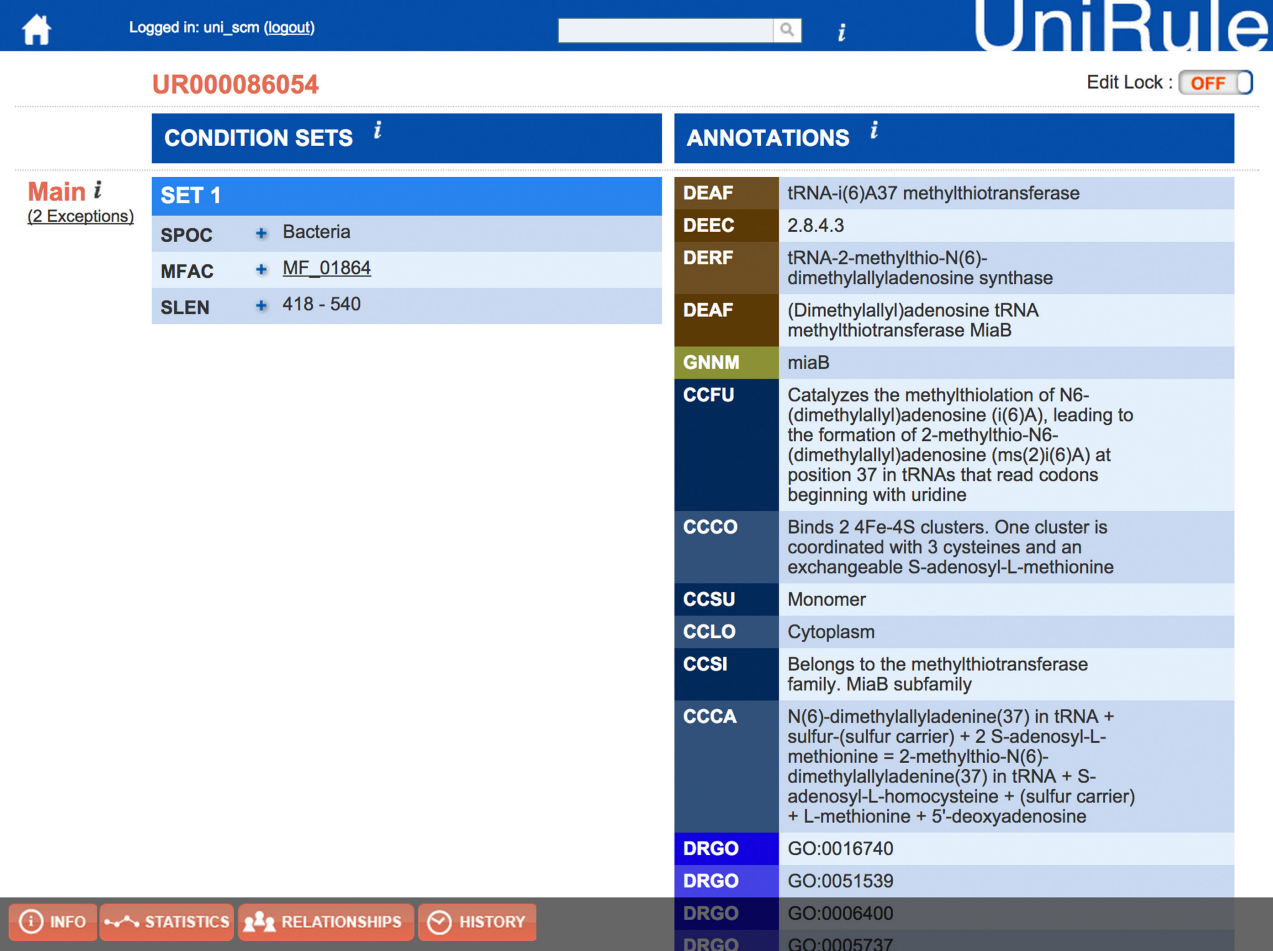
Representation of rule based on HAMAP entry MF_ 01864.

UniProt has developed two complementary rule-based systems to automatically annotate uncharacterized protein sequences of UniProtKB/TrEMBL. These are UniRule, in which rules are created as part of the process of expert curation of UniProtKB/Swiss-Prot, and SAAS, in which rules are derived automatically from UniProtKB/Swiss-Prot entries sharing common annotations and characteristics. Both UniRules and SAAS use the hierarchical InterPro classification of protein family and domain signatures (15) as a basis for protein classification and functional annotation. These rules share a common syntax that specifies annotations—including protein nomenclature, function and important residues—and necessary conditions, such as the requirement for conserved functional residues and motifs. InterPro integrates signatures from the HAMAP (16) and PIRSF (17) projects within the UniProt consortium. The creation of family signatures in HAMAP and PIRSF is tightly linked to the expert curation of literature characterized template entries in UniProtKB/Swiss-Prot, which allows highly specific functional annotation even within large and functionally diverse superfamilies. As an example the HAMAP signature MF_01864 see Figure 4, which encapsulates the information from only seven peer-reviewed publications covering four experimentally characterized proteins that serve as templates to annotate the function of the bacterial tRNA-2-methylthio-N(6)-dimethylallyladenosine synthase family to over 11 000 UniProtKB/TrEMBL records. The UniRule pipeline also leverages the manual curation of UniProtKB/Swiss-Prot for the continuous validation of rules: annotations are refreshed at each release of UniProtKB/TrEMBL, and the consistency of each rule evaluated by comparing the predicted annotations with those of the current version of UniProtKB/Swiss-Prot. Only those rules whose predictions perfectly match UniProtKB/Swiss-Prot are retained for the current production cycle.

**Figure 5.**
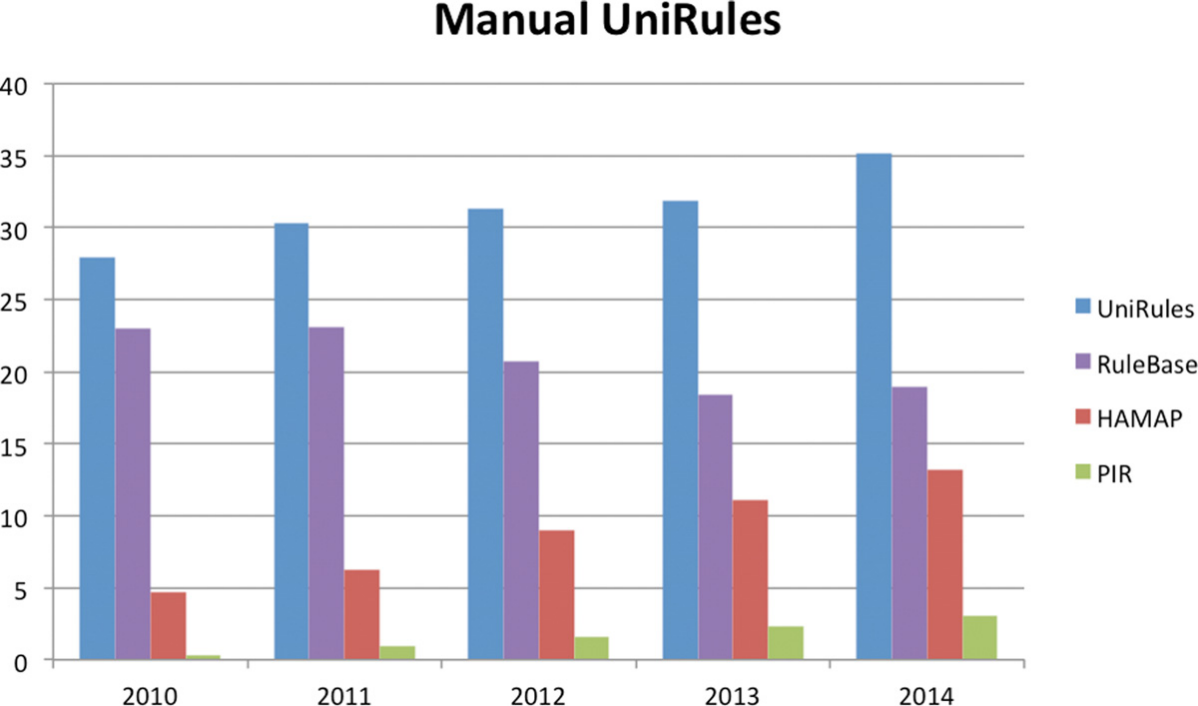
Growth of coverage of UniProtKB/TrEMBL by manually curated UniRules. The *Y*-axis shows the percentage coverage of UniProtKB/TrEMBL by UniRule as a whole as well as by the individual sources. PIR represents the combination of both PIR Site Rules (PIRSR) and PIR Name Rules (PIRNR).

The coverage of UniProtKB/TrEMBL has grown from 28% to 35% over the last 4 years despite the exponential increase in the size of the database, see Figure 5. This is due in part to a large amount of the increase being the result of the integration of redundant complete bacterial proteomes, which have been annotated by our existing UniRules for bacteria. Our strategy for addressing this redundancy will be discussed in the proteome section in this paper. Our automatic annotation priorities for UniRule generation are (i) to focus on using and annotating new functional data of interest for proteomes, such as enzymes and pathways and (ii) to expand our coverage into new taxonomic and protein families and (iii) to expand the scope of annotations by leveraging curated data via collaboration with external groups, as is the case with post-translational modification (PTM) data in RESID database (as an example see UniRule annotation of (UniProt F2I0T3) in the PTM/Processing section, with rule information based on RESID:AA0120).

### New website

We have redesigned the UniProt website following a user-centred design process, involving over 250 users worldwide with varied research backgrounds and use cases. User centred design is a design approach that is grounded in the requirements and expectations of users. They are included at every stage of the process, from gathering requirements to testing the end product. There were four main stages in the redesign. First, we began by analysing the current site with users through usability testing and gathering requirements through user workshops. This defined the aims of the redesign. We then worked on the information architecture and design to find possible solutions for the requirements and to address usability issues we had identified. We tested these designs with users starting from very early stages, using paper prototypes and sketches. This allowed us to iterate the designs rapidly according to user feedback in the third stage of the redesign and to develop the designs into high specification prototypes. In the final stage we implemented these designs, relying on user feedback to validate design decisions. The new UniProt website is now available.

**Figure 6.**
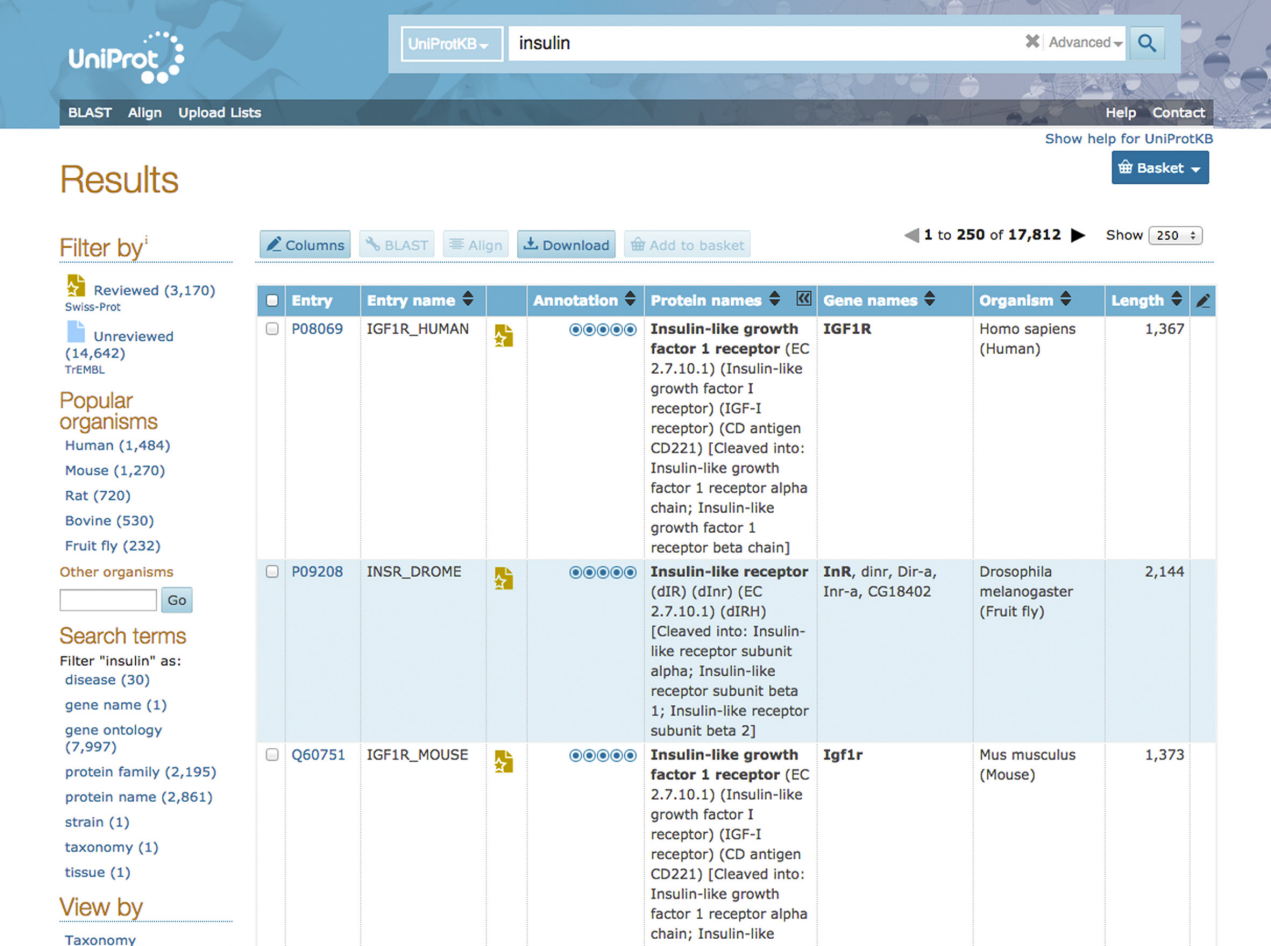
Customizable UniProt search results for ‘insulin’, with search term filters and breakdown by popular organism, and an additional column showing the annotation score for each entry.

We focussed primarily on creating easier navigation, improving the visibility and usability of existing functionality and structuring annotation data better for improved findability. The UniProt home page presents tiles for each data set to allow quick access, links to tools and help for users. All search result pages now include filters to help users narrow down their results, baskets to save data for later analysis and options to customize columns in the results table, see Figure 6.

**Figure 7.**
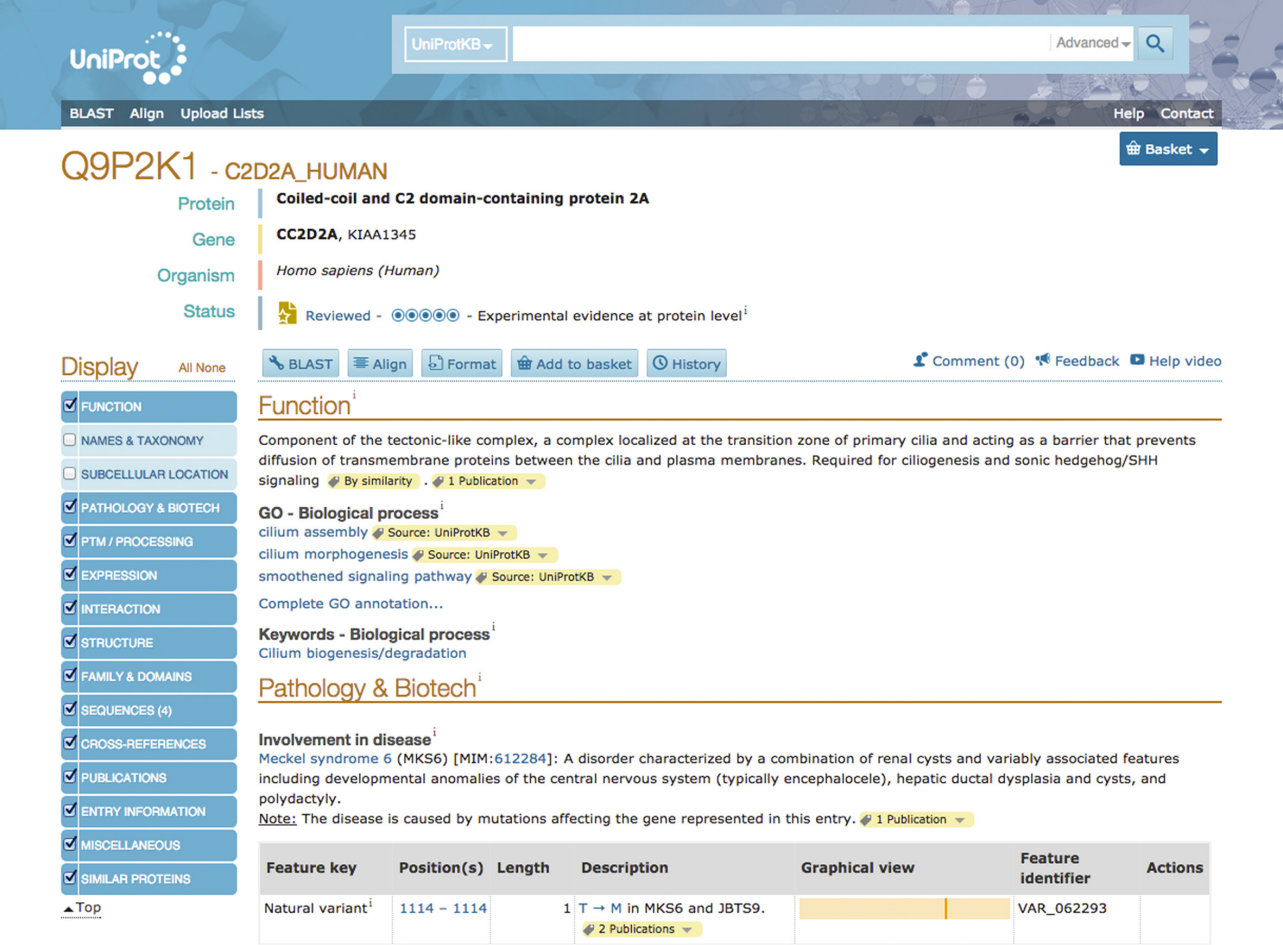
UniProt entry view for human coiled-coil and C2 domain-containing protein 2A (UniProt Q9P2K1).

The UniProt protein entry has been restructured to better tie together related annotations and data. New intuitive headings have been introduced such as Function, Subcellular location, Pathology & biotech, Interaction, etc. (see Figure 7).

**Figure 8.**
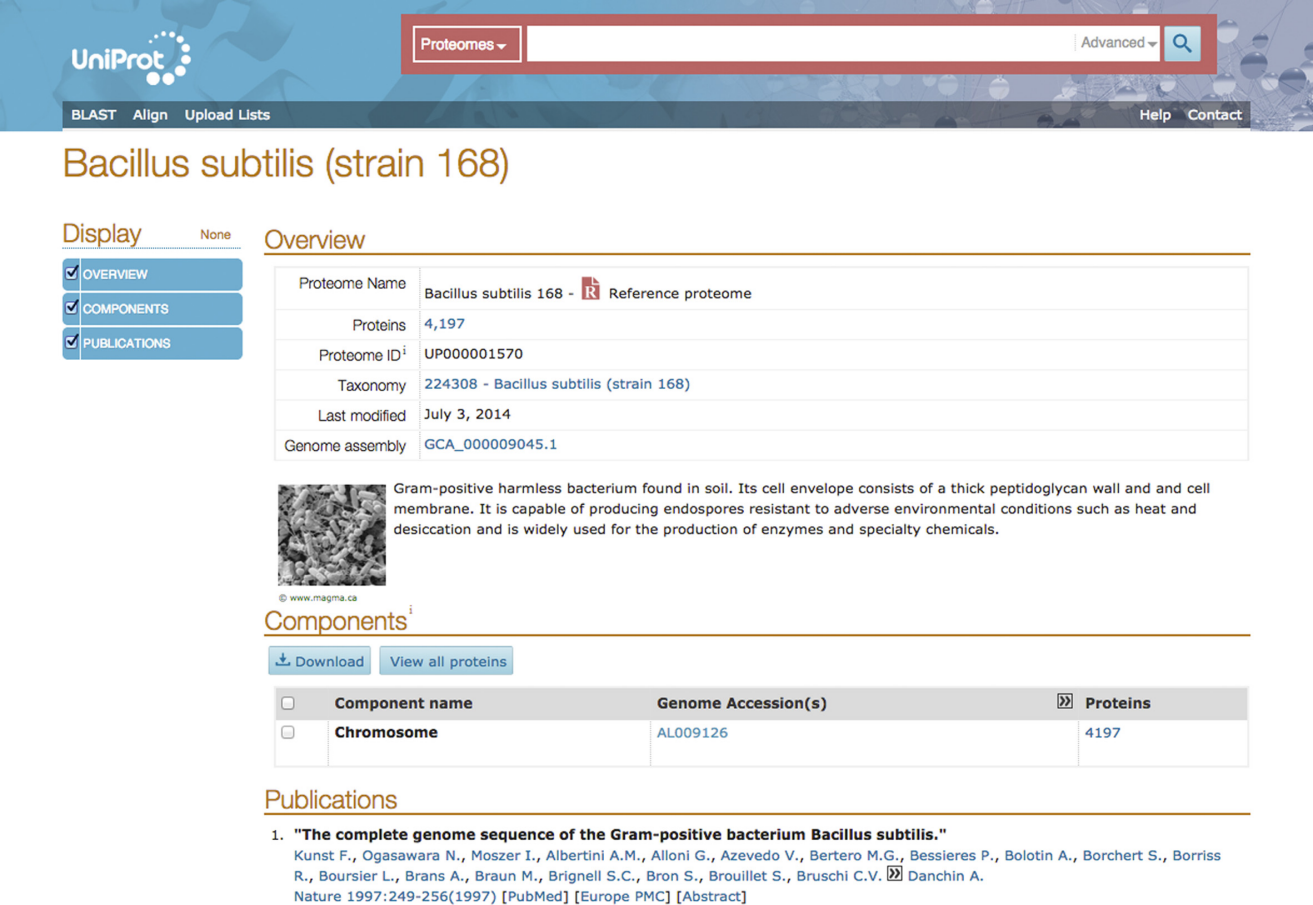
Reference proteome page for *Bacillus subtilis* (strain 168).

We have also added new pages for protein sets from completely sequenced organisms under the Proteomes data set, see Figure 8. This allows users to find proteomes and reference proteomes for their species of interest and download the data completely or select based on chromosome/plasmid.

Results pages for tools also present enhanced functionality, including Align for multiple alignments, Blast for sequence similarity search and Upload lists for retrieving batches or mapping identifiers to and from UniProt. Contextual help is available on all pages and links to UniProt help videos from the UniProt YouTube channel https://www.youtube.com/user/uniprotvideos.

Feedback from the beta site through the helpdesk and through direct testing with users has demonstrated a much improved user experience. We are continuing to follow the user-centred methodology to ensure that users can make the most of all our future developments. The user community can contact UniProt with feedback and queries through the Contact link on the website and they can also subscribe to our twitter feed @UniProt, follow our Facebook page or follow our blog Inside UniProt for the latest updates.

### Annotation scores

We have established a heuristic measure of the annotation content of a UniProt entry, called annotation score, to facilitate the identification of well-characterized proteins that are most informative for comparative analysis. The annotation score is computed in the following way (see http://www.uniprot.org/help/annotation_score for details): Different annotation types (e.g. protein names, gene names, functional and sequence annotations, GO annotations, cross-references) are scored either by presence or by number of occurrences. Annotations with experimental evidence score higher than equivalent inferred or predicted annotations, thereby favouring expert literature-based curation over automatic annotation. The score of an individual entry is the sum of the scores of its annotations. The score of a proteome is the sum of the scores of the entries that are part of the proteome. The open-ended interval obtained for these absolute numbers is translated into a 5-point-system by splitting it into 5 subintervals. Scores in the first interval are represented by ‘1 point out of 5’, those in the second by ‘2 points out of 5’, etc. An annotation score of 5 points is therefore associated with the best-annotated entries and a 1-point score denotes an entry with rather basic annotation.

We use the annotation score to determine the representative member of a UniRef cluster and also for the automatic selection of a reference proteome from a cluster of highly similar proteomes. The scores are also available on the website to give users a quick overview of the relative level of annotation of the UniProt entries in a search results.

### Usage of UniProt in the literature

**Figure 9.**
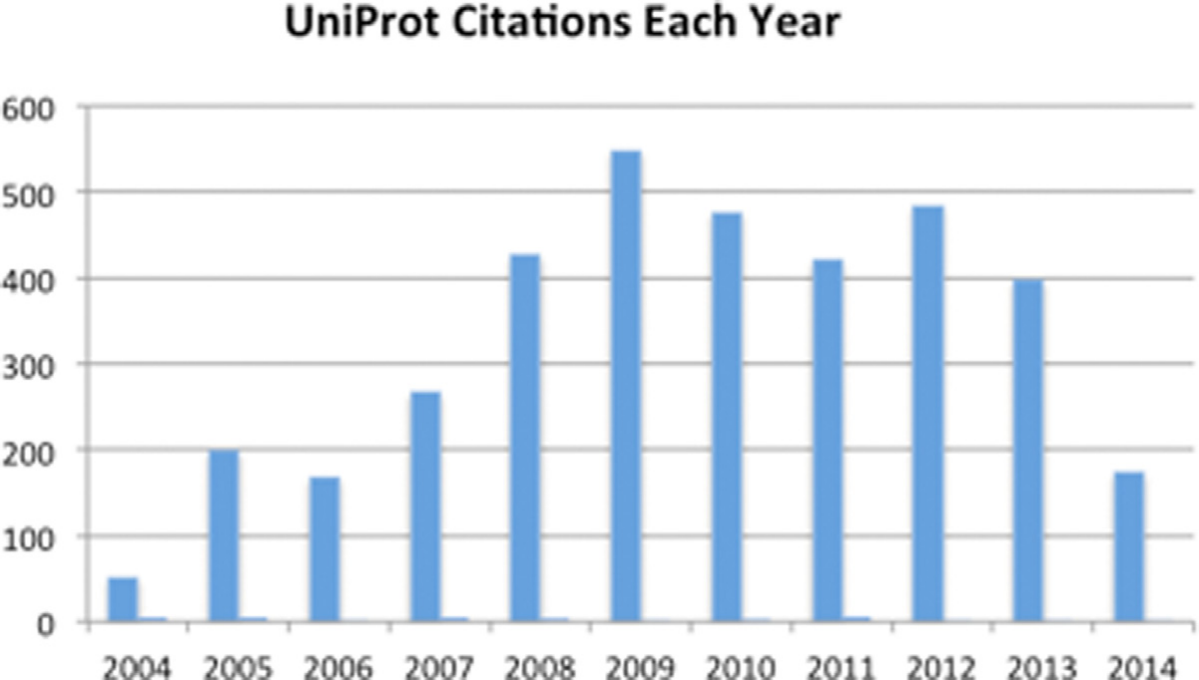
Distribution of citations to UniProt Publications by year.

**Figure 10.**
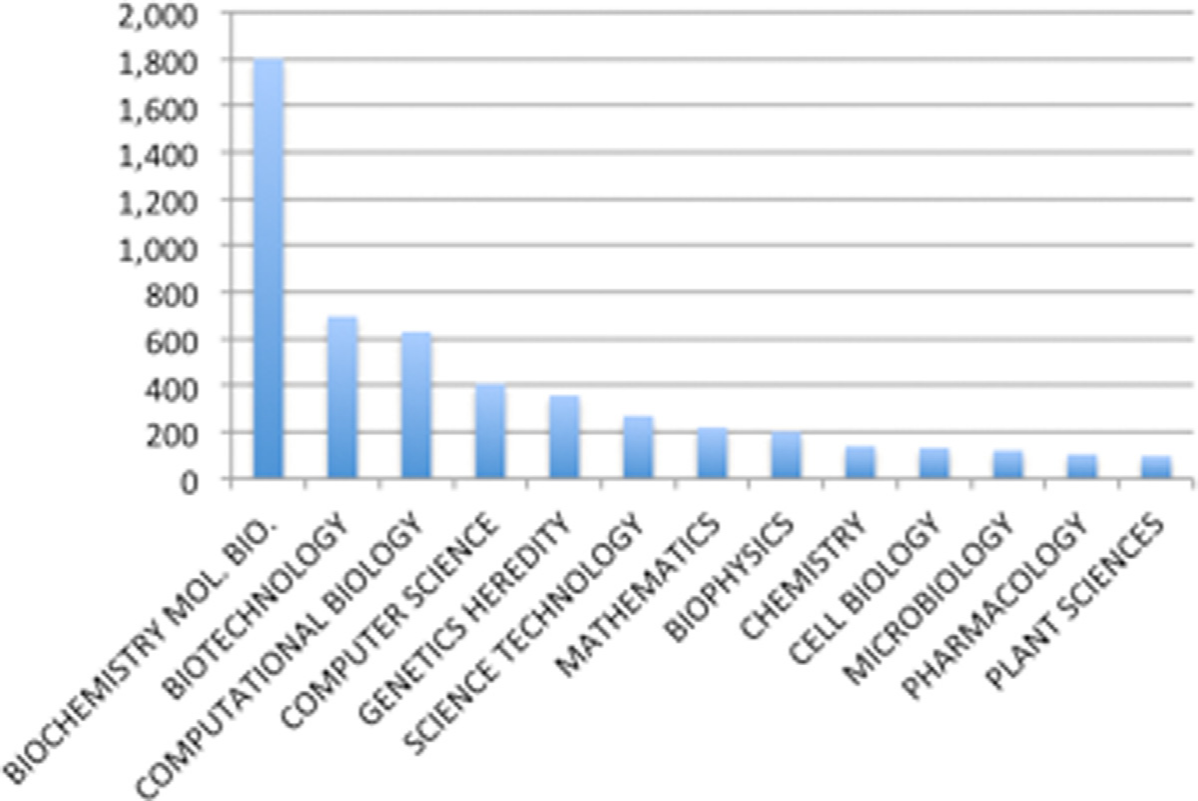
Distribution of number of publications citing UniProt, according to research categories. Note a publication may be classified in more than one category.

To gauge UniProt's impact on the research community we analysed the scientific literature citing UniProt Consortium publications (a set of 38 publications) in the time period 2002–14 with the aid of Thompson Reuters Web of Science tools. Overall UniProt publications were cited 3576 times in 898 unique journal titles. The distribution of citations per year is shown in Figure 9. The main research areas that these publications covered based on the indexing categories provided by Web of Science included Biochemistry and Molecular biology (over 50%), Biotechnology, Computational biology, Computer Science and Genetics, among others (see Figure 10). We also looked at the ISI 5 year Journal impact factor for this set of articles citing UniProt. The median score for the journals with 10 or more publications citing UniProt is 4.3. Note that there were 48 publications with impact factor over 20. These results illustrate that UniProt is widely used in broad areas of biomedical research, from algorithm development (using UniProt annotations as data sets, or the sequences), to resource building (integrating data from UniProt or providing links to UniProt data) and to protein identification, functional annotation and comparative studies. The citation numbers are an undercount as we have noted many examples where UniProt or other widely known resources are either not cited or cited in a way that was not recorded in the citation database. For example, a recent publication in the journal Cell made extensive use of UniProt annotation in defining a nuclear import pathway for ankyrin repeat containing proteins (18). Although UniProt was clearly mentioned throughout in the article text it was only cited in supplemental material, which is not included in many citation-tracking services.

## CONCLUSIONS

The past year has seen numerous important changes for UniProt. In particular many changes, such as the expansion of accession numbers, have been necessary to cope with the increase in sequences. However, we have several strategies to help our users deal with the deluge of protein data, such as the inclusion of proteome identifiers and the addition of further reference proteomes, to better navigate the deluge of new sequencing data. The provision of annotation scores will help users identify the proteins with the highest level of functional characterization, which should greatly aid comparative protein sequence analysis. We are particularly pleased to have released a completely redeveloped website that has been designed with the primary goal of enhancing the user's experience as they navigate our data. As well as these new developments we continue to focus upon our core mission to extract and organize experimental information on proteins from the literature and thus help scientists around the world to make further important discoveries. We encourage all our users to give us feedback on our data and website and to contact us via the e-mail help@uniprot.org, through the web at http://www.uniprot.org/contact or through our social media channels.

## SUPPLEMENTARY DATA

Supplementary Data are available at NAR Online.
